# Nuclear pore links Fob1‐dependent rDNA damage relocation to lifespan control

**DOI:** 10.1002/2211-5463.70193

**Published:** 2026-01-19

**Authors:** Yamato Okada, Mina Iwaki, Kyosuke Hagiri, Rei Izumi, Masahiko Harata, Chihiro Horigome

**Affiliations:** ^1^ Laboratory of Molecular Biochemistry, Graduate School of Agricultural Science Tohoku University Sendai Miyagi Japan; ^2^ Laboratory of Molecular Biochemistry, Faculty of Agriculture Tohoku University Sendai Miyagi Japan

**Keywords:** Fob1, genome stability, lifespan, nuclear pore, ribosomal RNA gene (rDNA), *Saccharomyces cerevisiae*

## Abstract

In budding yeast, the replication fork blocking protein Fob1 arrests replication forks at the ribosomal RNA gene (rDNA) locus, leading to DNA double‐strand breaks that promote genomic instability and limit replicative lifespan. rDNA damage has been reported to drive exit from the nucleolus, and persistent double‐strand breaks can relocate to the nuclear periphery, but how these spatial transitions are organized and how they influence genome stability and aging remain unclear. Here, we analyze the subnuclear localization of a site‐specific rDNA break and its functional relationship with nuclear pores. Using quantitative fluorescence microscopy, we show that damaged rDNA accumulates at the nucleolar–nucleoplasmic interface adjacent to the nuclear envelope. This position represents the minimal movement required to leave the nucleolar interior while maintaining contact with the nuclear periphery, in a manner reminiscent of nucleolar caps of higher eukaryotes. Cells defective in nuclear pore association display pronounced rDNA instability that is largely, but not completely, suppressed by deletion of Fob1, with partial restoration of rDNA stability. Disruption of nuclear pore association also shortens replicative lifespan, and this defect is partially rescued by Fob1 deletion, indicating that nuclear pores affect longevity through both Fob1‐dependent and Fob1‐independent pathways. These findings refine current models of rDNA damage handling in budding yeast and support a role for nuclear pores in spatially organizing Fob1‐induced rDNA damage to maintain rDNA stability and replicative lifespan.

## Graphical abstract

Damaged rDNA accumulates at a specific perinuclear interface that couples nucleolar escape with nuclear envelope association. Nuclear pores at this site help inhibit Fob1‐induced rDNA instability. This spatial organization of damage handling supports a functional link between nuclear architecture, rDNA stability, and replicative lifespan in yeast.

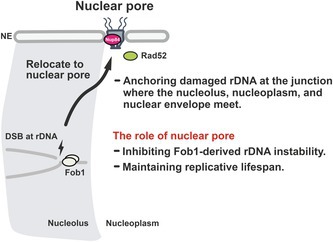

**Abbreviations**
ATM: Ataxia‐telangiectasia mutated
BIR: break‐induced replication
Chr. XII/Chr. III: Chromosome XII/Chromosome III
DAPI: 4′,6‐diamidino‐2‐phenylindole
DDR: DNA damage response
DSB: DNA double‐strand break
HR: homologous recombination
*lacO*/LacI: lactose operator/lactose repressor
MRN complex: Mre11‐Rad50‐Nbs1 complex
PFGE: pulsed‐field gel electrophoresis
rDNA: ribosomal RNA gene
RFB: replication fork barrier
RLS: replicative lifespan
rRNA: ribosomal RNA
*tetO*/TetI: tetracycline operator/tetracycline repressor

DNA double‐strand breaks (DSBs) represent one of the most serious forms of DNA damage. Incomplete or incorrect repair can result in chromosome fragmentation, translocations, and copy number alterations, leading to cellular aging, tumorigenesis, or cell death [1, 2]. In budding yeast (*S. cerevisiae*), DSB induction increases chromatin mobility [3, 4], and the hardly repaired‐damaged locus often relocates to the nuclear periphery, where it associates with the nuclear pore Nup84‐subcomplex and the inner nuclear membrane SUN‐domain protein Mps3 [5, 6, 7, 8, 9]. This relocation is regulated by DNA damage response kinases, chromatin remodelers, histone variant H2A.Z, and protein SUMOylation. Tethering to the nuclear envelope strongly influences repair outcomes: Binding to Mps3 inhibits ectopic recombination by limiting contact with other chromosomal regions, whereas association with nuclear pores promotes break‐induced replication (BIR) and microhomology‐mediated end‐joining (MMEJ) through the activity of SUMO‐targeted ligase Slx5/8 [5, 6, 7, 9, 10, 11].

Such subnuclear organization is conserved across eukaryotes. In *Drosophila*, heterochromatic DSBs escape from the domain and tether to the nuclear envelope, preventing ectopic recombination and enabling homologous recombination (HR) [12, 13, 14]. This tethering is particularly critical in repetitive regions, including heterochromatin, where recombination between non‐allelic repeats can cause large‐scale deletions or copy number changes that threaten genome integrity. By relocating breaks to the nuclear periphery, cells minimize the risks and bias repair toward more accurate HR. A similar mechanism operates in ribosomal RNA genes (rDNA), a large heterochromatic domain composed of giant repetitive sequences. In budding yeast, rDNA breaks exit the nucleolus, relocate to the nuclear pore, and are stabilized [15, 16]. This relocation separates breaks from intact repeats and is thought to facilitate access to recombination proteins normally excluded from the nucleolus.

In human cells, damaged rDNA also moves from the nucleolar interior to its periphery, forming structures known as ‘nucleolar caps’ [17]. Within the caps, RNA Polymerase I is suppressed and DNA damage response (DDR) proteins such as Rad51 (involved in HR) and ATM accumulate, supporting efficient HR. Furthermore, recent advances in super‐resolution microscopy have revealed that nuclear envelope–DNA damage coupling also occurs in human and mouse cells [18, 19, 20]. Notably, in these organisms the nuclear envelope actively invaginates toward damaged DNA, assisted by cytoplasmic microtubule‐driven tubules called dsbNETs. These observations expand the view of nuclear envelope–DNA damage coupling as a conserved mechanism.

rDNA instability is a major determinant of replicative lifespan (RLS) in yeast [21]. The rDNA locus is particularly prone to instability, as it consists of more than 100 tandem repeats that form the structural core of the nucleolus in eukaryotes [22, 23]. In budding yeast, the rDNA exists on chromosome XII as an array of approximately 150 repeating units that encode the 5S and 35S rRNA genes (Fig. S1). Its repetitive and highly active nature makes it a major hotspot for DSBs [24]. A key source of these breaks is replication fork stalling at the replication fork barrier (RFB), mediated by the DNA‐binding protein Fob1 [25, 26]. Loss of Fob1 suppresses fork stalling, restores rDNA stability, and extends cellular lifespan [21, 27].

Despite these insights into Fob1‐dependent replication stress, how cells spatially manage and repair such rDNA damage has remained unclear. Earlier studies independently described two distinct events following rDNA damage in yeast—the exit of damaged rDNA from the nucleolus to the nucleoplasm and its association with nuclear pores—but how these processes are connected and where the damaged rDNA resides during repair has not been defined. In this study, we precisely identify its destination as the nucleolar–nucleoplasmic interface adjacent to the nuclear envelope, representing the minimal and spatially organized relocation that simultaneously achieves both nucleolar segregation and nuclear pore association. Furthermore, we show that the nuclear pore association acts as an anti‐aging mechanism that stabilizes Fob1‐induced rDNA instability and resulting lifespan shortening. Our findings thus unify previously separate observations into a single mechanistic framework, establishing a direct link between nuclear pore function, rDNA maintenance, and replicative lifespan control.

## Materials and methods

### Plasmids, yeast strains, and growth conditions

Yeast strains used in this study are listed in Table S1. Cells were cultured at 30 °C in YPD medium for routine growth. Synthetic complete (SC) medium lacking the appropriate amino acids was used for selection of auxotrophic markers [28]. When required, G418 (Sigma‐Aldrich, Saint Louis, MO, USA) or clonNAT (WERNER BioAgents, Meisenweg, Jena, Germany) was added at a final concentration of 500 μg·mL^−1^ and 100 μg·mL^−1^, respectively. Yeast transformation was performed as previously described and verified by PCR and phenotypic analysis.

To visualize HO‐mediated DSB localization at the *MAT* locus, a *lacO* array (256 × binding sites) was integrated 4.4 kb from the cleavage sites [5]. For I‐*Sce*I‐mediated DSB localization, a *tetO* array (224 × binding sites) was inserted near the cleavage site within the rDNA region [15]. These repeat numbers are inherently unstable across strains and are typically less than 256 and 224, respectively. Expression of HO and I‐*Sce*I endonucleases was induced by adding a final 2% galactose to synthetic medium containing 2% raffinose (SR).

For the microscopy and image analysis, yeast cells were precultured at 30 ° C for 2 days on selective synthetic medium containing 2% glucose (SD). Cells were then inoculated into SR and grown overnight. On the following day, cultures were diluted into fresh SR and grown to exponential phase, reaching a density around 0.6–1.0 × 10^7^ cells·mL^−1^.

### Microscopy analysis

Fluorescence microscopy was performed as previously described [29]. Cells were harvested, fixed with 4% paraformaldehyde, and washed twice with PBS. Fluorescent images were acquired using an inverted microscope system consisting of an Olympus IX83 microscope, Olympus UPlanSApo 100 ×/ 1.40na oil objective and ORCA‐Flash4.0 V2 Digital CMOS camera C11440‐22CU (Hamamatsu, Hamamatsu City, Shizuoka, Japan). Florescent signals were analyzed using z‐stacks consisting of 23 optical sections at 0.25 μm intervals. For cell‐cycle staging, cells were classified as G1 (unbudded) or S/G2 (budded) based on bud morphology and the position and shape of nucleus, as previously described [30].

### HO and I‐ *Sce*I cleavage efficiency

DSB induction efficiency was assessed by quantitative PCR (qPCR) using CFX Connect Real‐Time PCR Detection System (Bio‐Rad, Hercules, CA, USA) and THUNDERBIRD® Next SYBR™ qPCR Mix (TOYOBO, Umeda, Osaka, Japan), as previously described [31]. qPCR was performed using primer sets across the DSB site and the control locus *SMC2*. Values were normalized to the uncut *SMC2* locus. Primer sequences are available in Table S2, and cut efficiencies are presented in Table S3.

### Pulsed‐field gel electrophoresis (PFGE)

PFGE samples were prepared as previously described [32]. Electrophoresis was performed in a 1.0% agarose gel with 0.5 × Tris‐borate‐EDTA (TBE) buffer and a CHEF‐MAPPER system (Bio‐Rad). Running conditions were 300–900 s pulse times at 100 V for 68 h at 14 °C. To quantify rDNA instability, signal intensities of chromosomes XII and IV were measured from GelRed®‐stained gel using ImageJ (Fiji). The intensity of Chr. XII was normalized to that of Chr. IV, which was assumed to remain constant across strains. Broad, unstable bands or structurally aberrant chromosomes may reduce signal intensity either due to dispersion within the gel or failure to enter the gel. Thus, the ratio of Chr. XII/Chr. IV reflects the relative stability of the rDNA locus.

### Replicative lifespan (RLS) analysis

Replicative lifespan was measured as previously described [33]. Briefly, yeast strains were streaked onto YPD plates and incubated overnight at 30 °C. The following day, single cells were then transferred onto fresh YPD plates using a micromanipulation system (Olympus BX43 equipped with a NARISHIGE (Minami‐Karasuyama, Tokyo, Japan) hydraulic micromanipulator) and incubated at 30 °C. Cells that emerged as small buds were relocated to separate positions, and their daughter cells were removed at each division. When the assay was paused, plates were stored at 4 °C. Monitoring continued until mother cells stopped dividing. If no division was observed in six consecutive inspections, the mother cell was considered non‐viable. The total number of budded daughter cells was recorded as the replicative lifespan of each mother cell.

## Results

### Damaged rDNA relocates to the nucleolar–nucleoplasmic interface at the nuclear envelope

**Fig. 1:**
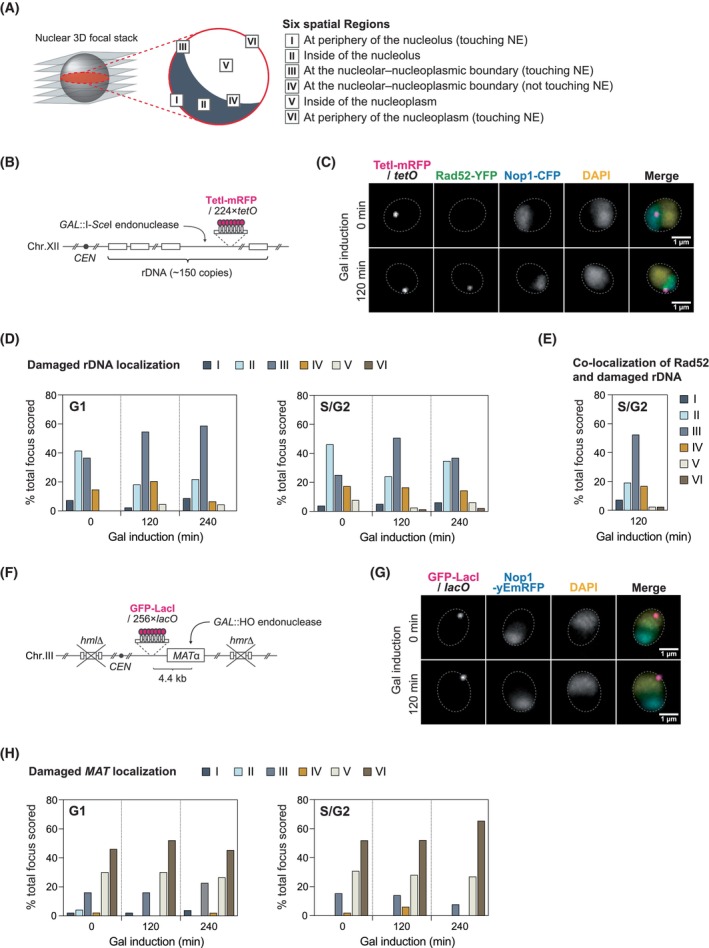
Damaged rDNA contacts the junction where the nucleolus, nucleoplasm, and nuclear envelope meet. (A) Locus positions were classified into six regions based on their spatial relationship to the nucleolus and the nucleoplasm using image stack analysis. NE stands for nuclear envelope. (B) Schematic of Chromosome XII (Chr. XII) in the indicated strain, harboring a *tetO* array adjacent to an I‐*Sce*I cut site within the rDNA, visualized by TetI‐mRFP. I‐*Sce*I was expressed under a galactose‐inducible promoter. (C) Representative images of the position of I‐*Sce*I cut site before and after galactose induction. Scale bar, 1 μm. (D) Localization of the I‐*Sce*I cut site in rDNA after 0, 120, and 240 min of galactose induction. The damaged site relocates to the nucleolar edge adjacent to the nuclear periphery in both G1 and S/G2 phases. Cells were classified as G1 (unbudded) or S/G2 (budded) based on bud morphology and the position and shape of nucleus. Cleavage efficiency, cell counts, and statistics are in Table S3. (E) Localization of rDNA cut site co‐localized with Rad52 foci after 120 min of galactose induction (based on panel d). (F) Schematic of Chromosome III (Chr. III) in the indicated strain, harboring a *lacO* array adjacent to the HO cut site at *MAT*, visualized by GFP‐LacI. HO was expressed under a galactose‐inducible promoter. (G) Representative images of *MAT* localization before and after galactose induction. Scale bar, 1 μm. (H) Localization of the HO cut site at *MAT* after 0, 120, and 240 min of galactose induction. Cleavage efficiency, cell counts, and statistics are in Table S3.

rDNA constitutes a major structural component of the nucleolus. To analyze in more detail the subnuclear localization of damaged rDNA, especially its relationship with the nuclear envelope and the nucleolus, which have been previously reported, we performed fluorescence microscopy, classifying the nucleus into six regions (Fig. 1A). To induce damage within the rDNA, we used a strain carrying a galactose‐inducible expression of the homing endonuclease I‐*Sce*I, which generates a unique DSB in the rDNA repeats (Fig. 1B). DSBs were visualized using TetI fused with mRFP bound to a *tetO* array integrated adjacent to the I‐*Sce*I recognition site (Fig. 1B,C) [15]. Nuclear compartments were identified with Nop1‐CFP as a nucleolar marker and DAPI staining, which primarily stains the nucleoplasm (Fig. 1C). Prior to DSB induction, mRFP foci were predominantly localized at the nucleolar center or at the nucleolar–nucleoplasmic boundary adjacent to the nuclear envelope (Fig. 1D, Regions II and III, respectively). In S/G2‐phase cells, Region II localization was particularly enriched, reaching 46.2% (Fig. 1D). Following 120–240 min of DSB induction, damaged rDNA accumulated primarily in Region III, at the nucleolar–nucleoplasmic boundary adjacent to the nuclear envelope (Fig. 1D). In the G1 phase, the localization to Region III consistently increased between 0 and 240 min after the break. However, in the S/G2 phase, it increased from 25% to 51% by 120 min, but subsequently decreased to 37% at 240 min. This is likely reflecting transient relocation and subsequent repair through replication‐associated pathways such as BIR, which displaces damaged or repairing rDNA from the nuclear periphery (Fig. 1D).

In higher eukaryotes, damaged rDNA relocates to the periphery of the nucleolus to form the reversible structures known as nucleolar caps. Nucleolar caps strongly repress RNA Pol I transcription and serve as a scaffold for the assembly of DDR proteins, including the Mre11‐Rad50‐Nbs1 (MRN) complex, ATM kinase, and homologous recombination factors such as Rad52 [17, 18]. Notably, nucleolar caps are frequently positioned near the nuclear envelope invaginations, functionally linking cap formation to perinuclear anchoring of damaged rDNA [18]. Although such caps have not been described in yeast, damaged rDNA in this organism also localizes to the nucleoplasm–nucleolus interface adjacent to the nuclear envelope, suggesting a conserved principle.

To test whether this localization facilitates access to DNA repair factors, particularly Rad52, a key homologous recombination protein enriched at DSBs outside the nucleolus. To test this, we examined the spatial relationship between damaged rDNA and Rad52 after DSB induction in S/G2 cells, based on the same biological sample set Fig. 1D. Rad52 was detected as YFP foci (Fig. 1C). Colocalization between rDNA and Rad52 was most frequent in Region III after 120 min of DSB induction, but the overall distribution paralleled that in Fig. 1D (Fig. 1E), indicating that Rad52 associates with DSBs mainly in the nucleoplasm, regardless of location. At 0 and 240 min, Rad52 foci were rarely observed, likely reflecting either the absence of damage or that the repair had progressed to a later‐stage recombination step (Table S3).

To compare spatial specificity of rDNA damage, we next analyzed the damage at the *MAT* locus. The locus was widely used in DSB localization studies, and it is known to relocate to the nuclear envelope upon break induction. In the strain, donor sequences at *HML* and *HMR* were deleted to prevent repair by gene conversion (Fig. 1F). Nuclear compartments were visualized with Nop1‐yEmRFP for the nucleolus and DAPI for nucleoplasm. A site‐specific DSB at *MAT* was induced by galactose‐driven expression of homing endonuclease HO, and the break site was visualized with a *lacO* array and GFP‐LacI system (Fig. 1F,G) [5]. Before DSB induction, the *MAT* locus primarily localized within the nucleoplasm or at the nuclear periphery on the non‐nucleolar side, with Regions V and VI being prominent (Region V: 30.0% in G1 and 30.8% in S/G2; Region VI: 46.0% in G1 and 51.9% in S/G2; Fig. 1H). After 120–240 min of DSB induction, the *MAT* locus remained at the same regions (Regions V and VI; Fig. 1H). This pattern indicated consistent periphery‐biased localization, whereas earlier studies reported no enrichment at the nuclear membrane prior to DSB induction [5, 9]. Such discrepancy may reflect methodological differences: Previous studies used nuclear pore component Nup49 as a periphery marker. To validate this, we applied the zoning assay in the same image set. Classifying DSBs into three equal zones relative to CFP‐tagged nuclear pore proteins confirmed transient enrichment of DSBs at the nuclear periphery (Zone 1) after 120–240 min of galactose induction in both G1 and S/G2 (Fig. S2a–d).

Together, these findings indicate that damaged rDNA moves from inside the nucleolus to the junction where the nucleolus, nucleoplasm, and nuclear envelope meet. This position represents the minimal movement required to achieve two previously observed events: nucleolar exit and binding to the nuclear envelope [15, 16].

### Fob1‐induced damage is a major cause of rDNA instability in *nup120*Δ

**Fig. 2:**
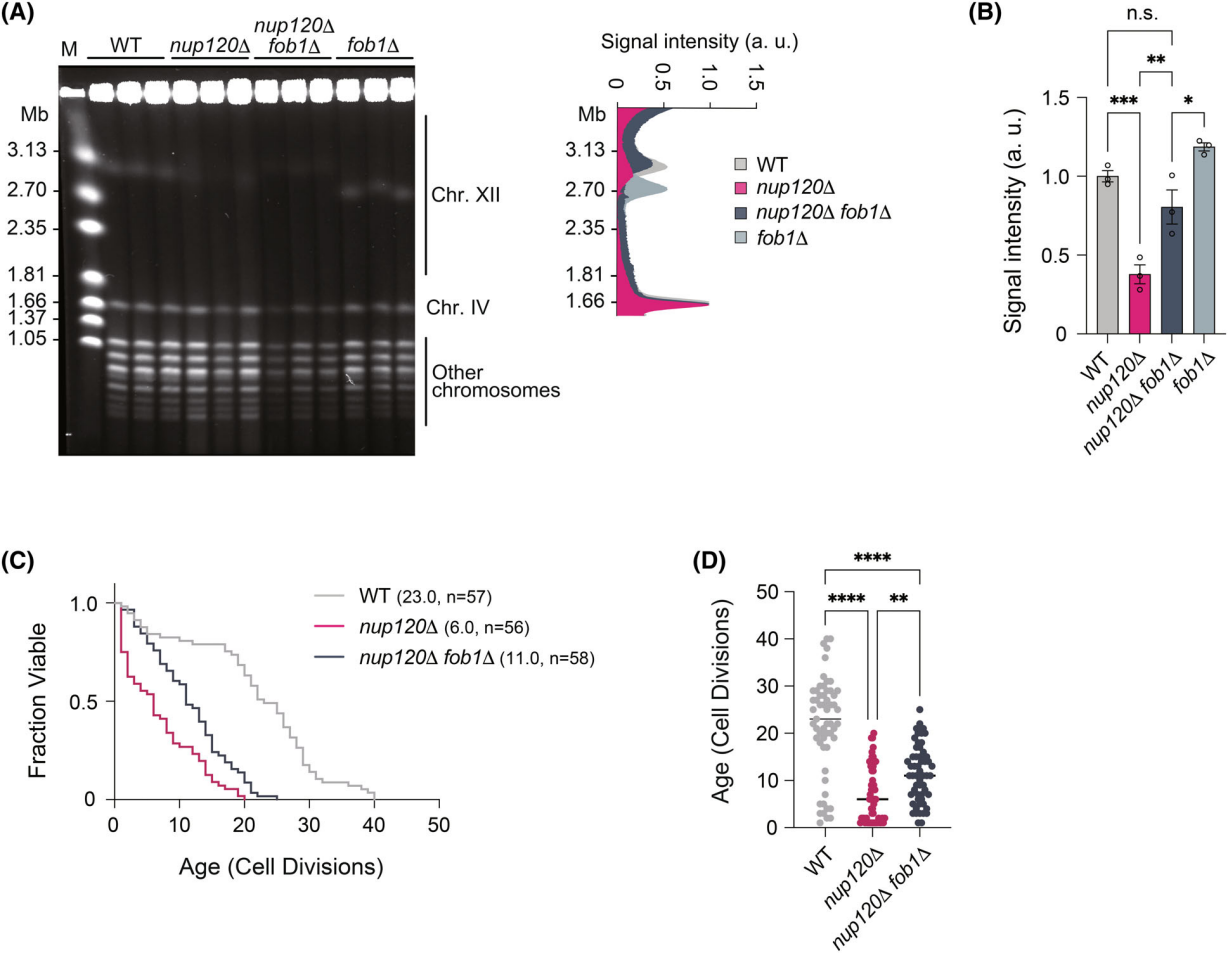
Fob1‐derived damage drives rDNA instability in *nup120*Δ. (A) Pulsed‐field gel electrophoresis (PFGE) was performed to assess rDNA stability in wild‐type (WT), *nup120*Δ, *nup120*Δ *fob1*Δ and *fob1*Δ strains. Gels were stained with GelRed® for visualization. DNA was extracted from cultures derived from single colonies. M indicates *Hansenula wingei* chromosomal DNA size markers. Signal intensity profiles (average of three lanes) are shown on the right. (B) Quantitation of rDNA instability (based on panel A). Chr. XII signal intensities were measured within a fixed square area matched to the size of the Chr. IV band, and then normalized to the intensity of Chr. IV as an internal control. Values are shown relative to the wild‐type strain. Data represent mean ± SEM from three independent colonies. Statistical significance was determined using ordinary one‐way ANOVA with Tukey's multiple comparisons test (*p* ≤ 0.05, *; *p* ≤ 0.01, **; *p* ≤ 0.001, ***; n.s., not significant vs. wild‐type). Statistics data are in Table S4. (C and D) Replicative lifespan curves of the indicated strains, median of cell divisions, and cell counts. Scatter plots of individual cell lifespans, where each point represents the number of divisions. Median lifespans are shown in parentheses. Statistical significance was determined by a Kruskal‐Wallis nonparametric test with Dunn's multiple comparisons test; (*p* ≤ 0.01, **; *p* ≤ 0.001, ***; *p* ≤ 0.0001, ****). Cell counts and statistics are in Table S4.

To investigate the physiological role of perinuclear localization that overlaps with the nucleolar–nucleoplasm boundary, we utilized the *nup120* deletion mutant, which impairs rDNA tethering to the nuclear pore. Previous work showed that *NUP120* disruption triggers rDNA destabilization [16]. Since rDNA instability is normally triggered by the activity of Fob1, we investigated whether the nuclear pore contributes to the repair of such damage. Fob1 binds the RFB and stalls replication progression unidirectionally, leading to DSB formation and rDNA instability [27, 34]. To test whether the rDNA instability observed in *nup120*Δ cells depends on Fob1, we performed pulsed‐field gel electrophoresis (PFGE) to resolve chromosome XII with their single and double mutations (Fig. 2A). As reported, *nup120*Δ cells displayed strong rDNA instability [16]. In contrast, the *nup120*Δ *fob1*Δ double mutant showed a clearly detectable Chr. XII band with partially restored signal intensity compared with the *nup120*Δ single mutant, although it did not reach the level of the wild‐type or *fob1*Δ single mutant (Fig. 2A,B). Because the stability in the double mutant was slightly lower than in the *fob1*Δ single mutant, these data suggest that most, but not all, of the rDNA instability in *nup120*Δ cells arises from Fob1‐induced DNA damage.

rDNA instability is a major determinant of RLS in yeast [21]. Stable rDNA maintenance promotes longevity, whereas instability shortens lifespan. To assess the impact of *NUP120* deletion on lifespan, we performed RLS analysis under standard conditions [33]. Wild‐type cells exhibited a median lifespan of approximately 23 generations (maximum ~40), whereas *nup120*Δ cells displayed a markedly shortened lifespan of around six generations (Fig. 2C). Deletion of *FOB1* in the *nup120*Δ background significantly recovered lifespan to 11 generations, reaching approximately half that of wild‐type (Fig. 2C). Although PFGE indicated that rDNA stability in the *nup120*Δ *fob1*Δ double mutant was partially restored relative to the *nup120*Δ single mutant (Fig. 2A,B), their lifespan remained moderately reduced, suggesting that Nup120 may contribute to longevity through additional pathways beyond rDNA maintenance. Given that rDNA instability in *nup120*Δ cells is largely dependent on Fob1 (Fig. 2A,B), we propose that nuclear pore association contributes to restoring Fob1‐induced rDNA instability, consistent with the established function of nuclear pores in safeguarding replication‐associated DNA damage [16, 35].

## Discussion

In this study, we show that damaged rDNA relocates to the nucleolar–nucleoplasmic interface in contact with the nuclear envelope. Furthermore, our results indicate that rDNA instability in *nup120*Δ cells is largely dependent on Fob1, suggesting that nuclear pore association contributes to restoring Fob1‐derived rDNA instability and thereby influences replicative lifespan.

The relocation of damaged rDNA to this specific perinuclear region points to a spatial mechanism that may promote accurate repair. This localization separates the damaged rDNA locus from the surrounding intact arrays within the nucleolus, thereby minimizing opportunities for ectopic recombination, while permitting access to Rad52, which resides exclusively in the nucleoplasm. The preferential accumulation in Region III, compared with Region VI that also borders the nucleoplasm, may reflect the coexistence of both segregation from the nucleolus and nuclear envelope tethering. This architecture resembles the nucleolar caps observed in higher eukaryotes, where the nuclear envelope invaginates toward a perinucleolar cap that supports homologous recombination. In our experiments, we did not observe clear nuclear envelope invaginations associated with rDNA DSBs in budding yeast, and such structures have not, to our knowledge, been systematically reported in this context. Our comparison with nucleolar caps is therefore intended to highlight a conceptual similarity rather than strict morphological equivalence, and our findings support a conserved principle of spatially organized repair at the nucleolar periphery.

Our results also support the view that the rDNA instability observed in *nup120*Δ cells is predominantly Fob1‐dependent, suggesting that nuclear pores provide a platform for resolving rDNA damage associated with replication fork arrest. Previous work has shown that nuclear pores contribute to the resolution of replication stress by facilitating fork restart and repair of collapsed forks [35]. Our findings extend this concept by demonstrating that rDNA as a major substrate of pore‐mediated stabilization. The improved stability in the *nup120*Δ *fob1*Δ double mutants highlight a role of nuclear pores in restoring Fob1‐initiated rDNA instability, while the residual instability compared to the *fob1*Δ single mutant suggests additional contributions to the repair of Fob1‐independent rDNA damage.

Finally, we show that the rDNA stabilizing function of nuclear pores influences replicative lifespan. Deletion of *NUP120* drastically shortened lifespan, but removal of *FOB1* partially rescued this defect. Lifespan did not recover fully to the wild‐type level, indicating that nuclear pores contribute to genome maintenance not only at rDNA but also at other genomic loci. Together, these findings support a model in which nuclear pores association helps restore Fob1‐induced rDNA instability and, in addition, contributes to Fob1‐independent pathways that affect genome stability and replicative lifespan. Although our study supports a functional link between nuclear pores, Fob1, rDNA stability, and lifespan, further work is required to investigate the molecular reactions occurring at the nuclear pore for these processes.

## Conflict of interest

The authors declare no conflict of interest.

## Author contributions

YO, MH, and CH conceived and designed the research. YO, MI, KH, and RI performed experiments, data curation and analysis. YO wrote the original draft. YO, MH, and CH reviewed and edited the manuscript, with inputs from all authors.

## Supporting information


**Fig. S1.** Structure of rDNA in *Saccharomyces cerevisiae*.
**Fig. S2**. Localization analysis of the *MAT* locus.
**Table S1**. List of yeast strains used in this study.
**Table S2**. List of primer pairs used in this study.
**Table S3**. Statistical analysis and replicative data of microscopy analysis.
**Table S4**. Statistical analysis and replicative data of PFGE and RLS assay.

## Data Availability

The data that support the findings of this study are available from the corresponding author [chihiro.horigome.b7@tohoku.ac.jp] upon reasonable request.
