# Supplementary material for: Nuclear pore links Fob1‐dependent rDNA damage relocation to lifespan control
Source: FEBS Open Bio. 2026 Jan 19;16(7):1305–13. doi: 10.1002/2211-5463.70193 (PMC13327006; doi:10.1002/2211-5463.70193)
Supplement: Supplementary file 1 — Fig. S1. Structure of rDNA in Saccharomyces cerevisiae. Fig. S2. Localization analysis of the MAT locus. Table S1. List of yeast strains used in this study. Table S2. List of primer pairs used in this study. Table S3. Statistical analysis and replicative data of microscopy analysis. Table S4. Statistical analysis and replicative data of PFGE and RLS assay. [file FEB4-16-1305-s001.pdf]

Supplementary Table S1. List of yeast strains used in this study.

Name	Genotype	Source
ML118-1D	*MAT***a** *ADE2 RAD52-YFP RDN25::* 224x*tetO-URA3-I-SceI-cs TetI-mRFP1-iYGL119W*	Torres-Rosell *et al*., 2007
YCH-255	derived from ML118-1D, pWJ1108 (pRS423-Gal1-10-I-*Sce* I) pWJ1529 (pRS415-*NOP1-CFP*)	Torres-Rosell *et al*., 2007
YCH-29	GA-6844; *MAT alfa*, *Δho hml::ADE1 hmr::ADE1 ade3::GALHO ade1-100 leu2-3, 112 lys5 trp1::hisG ura3-52 CFP-NUP49 GFP-LacI:LEU2 MAT::lacO* repeats:*TRP1*	Horigome *et al*., 2014
YCH-316	derived from GA-6844, *NOP1-yEmRFP-KanMX6*	This study
NOY408-1b (WT)	*MAT a leu2-3, 112 trp1-1 can1-100 ura3-1 ade2-1 his3-11,15*	Nogi *et al*,. 1991
YCH-554	derived from NOY408-1b, *nup120*Δ::*natMX*	This study
YCH-555	derived from NOY408-1b, *nup120*Δ::*natMX fob1*Δ::*LEU2*	This study
YCH-556	derived from NOY408-1b, *fob1*Δ::*LEU2*	This study

Supplementary Table S2. List of primer pairs used in this study.

Target	Sequence
SMC2	F: AATTGGATTTGGCTAAGCGTAATC
	R: CTCCAATGTCCCTCAAAATTTCTT
I-*Sce* I-mediated DSB (rDNA)	F: ACGGAACAGCTATGACCATGATTAC
	R: GGCCCGGTACATTACGCTAG
HO -mediated DSB (*MAT*)	F: GAATATGGGACTACTTCGCGCAAC
	R: CGTCACCACGTACTTCAGCATAA

Supplementary Table S3. Statistical analysis and replicative data of microscopy analysis.

Fig. 1d

min after I-*Sce* I	Cell cycle	% of intact I-*Sce* I	# of nuclei	% of cells in each Region					
				I	II	III	IV	V	VI
0	G1	100.00	41	7.3	41.5	36.6	14.6	0.0	0.0
0	S/G2	100.00	52	3.8	46.2	25.0	17.3	7.7	0.0
120	G1	39.88 ± 5.23	44	2.3	18.2	54.5	20.5	4.5	0.0
120	S/G2	39.88 ± 5.23	79	5.1	24.1	50.6	16.5	2.5	1.3
240	G1	37.55 ± 8.97	46	8.7	21.7	58.7	6.5	4.3	0.0
240	S/G2	37.55 ± 8.97	49	6.1	34.7	36.7	14.3	6.1	2.0

Fig. 1e (based on the same biological sample set as Fig. 1d)

min after I-*Sce* I	Cell cycle	% of intact I-*Sce* I	# of nuclei	% of cells in each Region					
				I	II	III	IV	V	VI
0	S/G2	100.00	3	0.0	33.3	66.7	0.0	0.0	0.0
120	S/G2	39.88 ± 5.23	42	7.1	19.0	52.4	16.7	2.4	2.4
240	S/G2	37.55 ± 8.97	8	12.5	12.5	37.5	12.5	12.5	12.5

Fig. 1h

min after HO	Cell cycle	% of intact HO	# of nuclei	% of cells in each Region					
				I	II	III	IV	V	VI
0	G1	100.00	50	2.0	4.0	16.0	2.0	30.0	46.0
0	S/G2	100.00	52	0.0	0.0	15.4	1.9	30.8	51.9
120	G1	14.32 ± 0.65	50	2.0	0.0	16.0	0.0	30.0	52.0
120	S/G2	14.32 ± 0.65	50	0.0	0.0	14.0	6.0	28.0	52.0
240	G1	7.24 ± 0.48	53	3.8	0.0	22.6	1.9	26.4	45.3
240	S/G2	7.24 ± 0.48	52	0.0	0.0	7.7	0.0	26.9	65.4

Fig. S2d (based on the same biological sample set as Fig. 1h)

min after HO	Cell cycle	% of intact HO	# of nuclei	% of cells in each zone			*P*- value	Statistical test
				1	2	3		
0	G1	100.00	50	30.0	16.0	54.0	0.0042	χ2 test comparing vs. random distribution (zone 3)
0	S/G2	100.00	50	30.0	22.0	48.0	0.0444	χ2 test comparing vs. random distribution (zone 3)
0	G1	100.00	50	30.0	16.0	54.0	1.4776	χ2 test comparing vs. random distribution (zone 1)
0	S/G2	100.00	50	30.0	22.0	48.0	1.4778	χ2 test comparing vs. random distribution (zone 1)
120	G1	14.32 ± 0.65	50	58.0	12.0	30.0	0.0006	χ2 test comparing vs. random distribution (zone 1)
120	S/G2	14.32 ± 0.65	50	22.0	18.0	28.0	0.2519	χ2 test comparing vs. random distribution (zone 1)
240	G1	7.24 ± 0.48	50	44.0	26.0	30.0	0.1512	χ2 test comparing vs. random distribution (zone 1)
240	S/G2	7.24 ± 0.48	50	48.0	40.0	24.0	0.0444	χ2 test comparing vs. random distribution (zone 1)

Supplementary Table S4. Statistical analysis and replicative data of PFGE and RLS assay.

Fig. 2b

Relevant genotype	Chr. XII intensity relative to WT	*P*-value	Statistical test
WT	1.00 ± 0.04	0.0007	Tukey's multiple comparisons test vs. WT and *nup120*Δ
*nup120*Δ	0.38 ± 0.06	0.2290	Tukey's multiple comparisons test vs. WT and *nup120*Δ *fob1*Δ
*nup120*Δ *fob1*Δ	0.80 ± 0.11	0.0076	Tukey's multiple comparisons test vs. *nup120*Δ and *nup120*Δ *fob1*Δ
*fob1*Δ	1.19 ± 0.03	0.0142	Tukey's multiple comparisons test vs. *nup120*Δ *fob1*Δ and *fob1*Δ

Fig. 2d

Relevant genotype	# of cells	*P*-value	Statistical test
WT	57	< 0.0001	Dunn’s multiple comparisons test vs. WT and *nup120*Δ
*nup120*Δ	56	0.0046	Dunn’s multiple comparisons test vs. *nup120*Δ and *nup120*Δ *fob1*Δ
*nup120*Δ *fob1*Δ	58	< 0.0001	Dunn’s multiple comparisons test vs. *nup120*Δ *fob1*Δ and WT
